# What can we say about the impact of teleconsultations on preventing psychiatric inpatient treatment during COVID-19 pandemic?

**DOI:** 10.1192/j.eurpsy.2022.1476

**Published:** 2022-09-01

**Authors:** I. Figueiredo, I. Cargaleiro, M. Nascimento

**Affiliations:** Centro Hospitalar Psiquiátrico de Lisboa, Clinic 3, Lisboa, Portugal

## Abstract

**Introduction:**

Telepsychiatry was proved effective and satisfactory in settings like the emergency department and mental health services, but its use is historically restricted. Although there are several studies about telepsychiatry pros and cons, more is needed to know about its effect on patient’s follow-up and its influence on inpatient treatment rates, specifically during COVID-19 pandemic.

**Objectives:**

The aim is to look for an eventual change on psychiatric inpatient admissions, during COVID-19 pandemic, when psychiatric patients are in follow-up through teleconsultation.

**Methods:**

We compared the number of hospitalizations for 3 different 6 months periods of time: the 2^nd^ semester of 2019 with no teleconsultations, March-August 2020 only with teleconsultations (except some few 1^st^ consultations) and the 1^st^ semester of 2021 with face-to-face and teleconsultations. A one-way repeated measures ANOVA was conducted on a 1050 patients sample.

**Results:**

The statists showed that the type of approach in consultations didn’t lead to statistically significant differences in hospitalizations (F test-statistic = 0.33086, p = 0.718345).

**Conclusions:**

There is a plethora of advantages about telepsychiatry and it was already shown to be as effective as in-person contact. Some articles show an association of telepsychiatry with a decrease in hospitalization rates, but mostly display similar clinical outcomes. In this study, the authors found that the results follow the latter tendency, although we must consider the COVID-19 pandemic as a possible decompensation and worsening clinical factor. More studies on this matter are important to better understand the potential benefits (and risks) of this treatment setting.

**Disclosure:**

No significant relationships.

